# Earthworm extract ameliorates colitis via modulation of the PI3K/AKT pathway: attenuation of inflammation and restoration of intestinal barrier integrity

**DOI:** 10.3389/fmed.2026.1787022

**Published:** 2026-05-29

**Authors:** Shaoru Zhang, Lihui Wang, Qi Shen, Luxi Shangguan, Ting Sheng, Yuqing Gong, Hongwei Wang, Ruofan Liao, Yang An, Yanting Wen, Shuan Wang, Xiaotian Chen, Yong Wang

**Affiliations:** 1 Department of Clinical Nutrition, Nanjing Drum Tower Hospital, Affiliated Hospital of Medical School, Nanjing University, Nanjing, China; 2 State Key Laboratory of Analytical Chemistry for Life Science & Jiangsu Key Laboratory of Molecular Medicine, Medical School, Nanjing University, Nanjing, China; 3 The People’s Hospital of Danyang & Affiliated Danyang Hospital of Nantong University, Danyang, China; 4 Danyang Center for Disease Control and Prevention, Danyang, China

**Keywords:** ulcerative colitis, earthworm extract, tight junction proteins, inflammation, Caco-2 cell

## Abstract

**Background:**

Ulcerative colitis (UC) is driven by a self-perpetuating cycle of intestinal barrier disruption and chronic inflammation. Current therapeutic options are limited, underscoring the need for safer and more effective agents. Earthworm extract (EE, derived from Dilong used in traditional Chinese medicine) has a documented history of use in inflammatory and reparative contexts. In this study, we evaluated the therapeutic potential and mechanism of EE using a murine model of colitis and Caco-2 intestinal epithelial cells model.

**Methods:**

A murine model of ulcerative colitis was established with 3% DSS. Serum cytokines were measured by ELISA, and intestinal permeability was assessed via the FITC-dextran assay. *In vitro*, LPS and TNF-*α*/IFN-*γ*-stimulated Caco-2 monolayers were used to monitor transepithelial electrical resistance. Gene and protein expression were analyzed by qRT-PCR, western blotting, immunohistochemistry, and immunofluorescence. Mechanisms were investigated through network pharmacology and validated by pharmacological intervention. The bioactive components in EE were characterized by heat inactivation, protease sensitivity, and dialysis-based molecular weight fractionation.

**Results:**

Earthworm extract administration significantly alleviated DSS-induced disease severity, including weight loss, colon shortening, and histological damage. At the molecular level, EE suppressed pro-inflammatory cytokines (TNF-α, IL-1β, and IL-6) by inhibiting the NF-κB pathway and enhanced intestinal barrier function by upregulating tight junction proteins (ZO-1 and Occludin) *in vivo*. These protective effects were corroborated *in vitro*, where EE restored epithelial monolayer integrity and reversed inflammatory cytokine release and tight junctional protein loss induced by LPS or TNF-α/IFN-γ. Through an integrative network pharmacology analysis, the PI3K/AKT pathway was identified as a key signaling hub mediating the effects of EE against UC. Subsequent experimental validation confirmed that EE significantly inhibits PI3K/AKT activation. Notably, EE alone exhibited no detectable short-term toxicity or adverse effects. Characterization studies confirmed that the bioactive components in EE are proteinaceous, heat-labile, non-dialyzable macromolecules.

**Conclusion:**

Our findings demonstrate that EE ameliorates experimental colitis primarily through inhibition of the PI3K/AKT signaling pathway, thereby attenuating inflammation and restoring intestinal barrier integrity, while maintaining a favorable safety profile. This work provides a scientific rationale for developing EE as a promising natural agent for inflammatory bowel disease.

## 1 Introduction

Ulcerative colitis (UC) is an inflammatory disease primarily affecting the colorectum, characterized by symptoms such as weight loss, abdominal pain, diarrhea, and hematochezia (1). The relapsing nature of UC not only impairs quality of life but also increases the risk of colorectal cancer (2, 3). Historically considered a disease of industrialized nations, UC has emerged as a global health concern in the 21st century, with rising incidence in newly industrialized countries and substantial economic burden worldwide (4, 5).

The pathogenesis of UC is multifactorial, involving impaired intestinal epithelial barrier function, disturbed immune response, and dysbiosis of the intestinal flora (4, 6). During UC progression, the expression of tight junction (TJ) proteins such as ZO-1, Occludin, and claudins is significantly downregulated, leading to increased intestinal permeability (7). This allows luminal pathogens to invade colonic tissue and activate pattern recognition receptors (PRRs) such as Toll-like receptors (TLRs), triggering downstream NF-κB and MAPK signaling cascades that drive pro-inflammatory cytokine production (8). This inflammation not only mediates tissue damage but also further impairs barrier repair, establishing a pathological feedback loop (9–12). Consequently, therapeutic approaches that concurrently target the inflammatory cascade (e.g., NF-κB, STAT3, or PI3K/AKT) and promote epithelial barrier restoration are considered promising for UC treatment (13, 14).

Currently, aminosalicylic acid drugs, corticosteroids, and immunosuppressants are mostly applied in the first-line clinical treatment of UC, but they have poor efficacy and severe side effects. In recent years, natural products derived from traditional medicine has become a new direction in the study of drugs for the treatment of UC due to its advantages, such as multi-target activity, favorable safety profiles, low price, and wide range of sources (15–17). Earthworm, known as “Dilong” in traditional Chinese medicine, has been documented in classical texts such as the *Compendium of Materia Medica* for its antithrombotic, tissue-reparative, and anti-inflammatory properties. Modern pharmacological studies indicate that earthworm extract (EE) exerts anti-inflammatory effects by inhibiting TLR4/NF-κB signaling, reducing the expression of TNF-α, IL-1β, and IL-6, and suppressing M1 macrophage polarization (18, 19). Additionally, EE promotes tissue repair by modulating the TGF-β/Smad pathway, enhancing collagen remodeling and angiogenesis in wound healing models (20, 21). However, its therapeutic potential and mechanism of action in UC – particularly whether EE modulates key pathways such as PI3K/AKT, JAK/STAT3, or AMPK to simultaneously suppress inflammation and restore barrier integrity – remain incompletely understood.

Therefore, this study aimed to systematically evaluate the protective effects of EE against UC and elucidate its underlying molecular mechanisms. We employed a well-established dextran sulfate sodium (DSS)-induced murine colitis model, which closely recapitulates key histopathological and clinical features of human UC and allows controllable disease induction (18–20). Through integrated *in vivo* and *in vitro* approaches, we assessed the impact of EE on inflammatory responses, epithelial barrier integrity, and related signaling pathways, with a particular focus on the PI3K/AKT pathway identified through network pharmacology analysis.

## 2 Materials and methods

### 2.1 EE preparation and analysis

Earthworm extract (EE) was prepared according to a previously established protocol (21). Briefly, live earthworms were rinsed and dissected to remove internal organs (including the entire intestinal tract and gizzard). The remaining body walls were then dried and ground into a fine powder. The powder (10 g) was then subjected to aqueous extraction by soaking in distilled water (100 mL) for 30 min, followed by ultrasonication for 40 min at 25 °C. After centrifugation, the supernatant was collected, and the residue was re-extracted under identical conditions. The combined supernatants were filtered, concentrated under reduced pressure at 50 °C using a rotary evaporator, and finally lyophilized to obtain the final EE powder (yield: 1.89 g). To eliminate potential endotoxin contamination, the crude EE powder was further treated with an endotoxin removal kit (Beyotime, Wuhan, China) prior to use (22). The endotoxin level in the treated EE was then determined using the Beyotime Chromogenic LAL Endotoxin Assay Kit following the manufacturer’s instructions (23).

For quality control purposes, the contents of characteristic nucleotides and their hydrolysates in EE were analyzed using HPLC, and fingerprint analysis was performed to assess batch-to-batch consistency, as described in the Supplementary material (21, 24, 25). To characterize the bioactive component(s) in the earthworm extract (EE), three independent approaches were employed: heat inactivation, protease sensitivity, and dialysis-based molecular weight fractionation. For heat inactivation, EE was incubated at 60, 80, and 100 °C for 30 min, immediately cooled on ice, and centrifuged to remove precipitates. For protease sensitivity, EE was incubated with Proteinase K (50 μg/mL) at 50 °C for 2 h, followed by heat inactivation at 95 °C for 10 min (26). For molecular weight fractionation, EE was dialyzed using a 3.5 kDa MWCO membrane (27). After dialysis, the retentate (containing molecules >3.5 kDa) and the dialysate (containing molecules <3.5 kDa) were collected separately.

### 2.2 Animal models construction

Dextran sulfate sodium (DSS, molecular weight 36,000–50,000) was purchased from MP Biomedicals (Santa Ana, CA, United States). The clinical anti-inflammatory drug mesalazine (Etiasa, Shanghai, China) served as a positive control in this study. Male C57BL/6 mice (6 weeks old) were supplied by the Institute of Model Animals at Nanjing University and maintained under specific pathogen-free (SPF) conditions with controlled temperature (22 ± 1 °C), humidity (50 ± 1%), and a 12 h light/dark cycle. After 1 week of acclimatization with free access to food and water, the experiments were initiated following approval from the Institutional Animal Care and Use Committee of Drum Tower Hospital, Nanjing University Medical School (Approval No. 2023AE01007).

The animals were randomly assigned to five groups (n = 6 per group): a normal control group (Ctrl), a DSS-induced colitis model group (DSS), a DSS plus mesalazine treatment group (DSS + M), a DSS plus EE treatment group (DSS + EE), and an EE-alone control group (EE). Colitis was induced by providing the mice with 3% DSS in their drinking water freely for 7 consecutive days (28), followed by a 5-day recovery period with plain sterile water. Previous studies have shown that minor variations in fluid intake do not significantly affect the severity of DSS-induced mucosal injury, as injury severity correlates with DSS concentration rather than total dose consumed (29). Concomitant with DSS exposure, mice in the DSS + EE and DSS + M groups received daily intragastric administration of EE (300 mg/kg) (21, 30) or mesalazine (200 mg/kg), respectively. Mice in the EE-alone group were gavaged with EE only, while the Ctrl and DSS groups received an equivalent volume of vehicle. This design separates the route of DSS induction (ad libitum drinking) from therapeutic intervention (gavage), ensuring precise dosing of EE without interfering with DSS consumption. Mice were housed in groups (3 mice per cage) and DSS solution was freshly prepared and replaced daily.

To investigate whether the therapeutic effect of EE depends on inhibition of the PI3K/AKT pathway, a PI3K-specific agonist (740Y-P, MCE, United States) was employed. C57BL/6 J mice were randomly divided into six groups (*n* = 6 per group): (1) Ctrl; (2) Ctrl+740Y-P; (3) DSS; (4) DSS + 740Y-P; (5) DSS + EE; and (6) DSS + EE + 740Y-P. 740Y-P was administered intraperitoneally at 10 mg/kg/day for 12 consecutive days. Mice in the Ctrl + 740Y-P group received 740Y-P alone, while those in the DSS + 740Y-P group received 740Y-P in combination with DSS treatment. In the DSS + EE+740Y-P group, following DSS exposure, mice were administered EE (300 mg/kg, p.o.) together with 740Y-P (10 mg/kg, i.p.) daily for 12 consecutive days (31, 32).

All treatments were synchronized throughout the experimental timeline. Body weight was monitored daily, and the presence of diarrhea and fecal blood was documented. At the endpoint, all mice were humanely euthanized via intraperitoneal injection of an overdose of sodium pentobarbital (240 mg/kg). The blood, colon, spleen, liver, kidney, heart, brain, and lung were harvested. Portions of these tissues were fixed in 4% paraformaldehyde (Servicebio, Wuhan, China) for paraffin embedding and subsequent histopathological examination. The remaining tissue samples were snap-frozen and stored at −80 °C for further molecular analyses.

### 2.3 Disease activity index (DAI)

The DAI was used to determine the weight loss percentage, fecal viscosity change and blood content in the stool of the mice. These three scores were subsequently summed and divided by 3 to evaluate the severity of colitis (15). The specific scoring criteria were as follows: for weight loss percentage, 0, 1–5%, 5–10%, 10–20% and greater than 20% were scored as 0, 1, 2, 3, and 4, respectively. Stool consistency was scored on a 0–4 scale: 0 for normal, well-formed pellets; 1 for soft, formed stools that adhered to the anus; 2 for semi-formed, loose stools; 3 for liquid stools without anal adherence; and 4 for profuse watery diarrhea. For the evaluation of bloody stool, the scoring was defined as: score 0 indicating the absence of blood in the stool; score 1 for slight presence of blood; score 2 for mild blood in the stool; score 3 for obvious blood presence; and score 4 for severe bleeding.

### 2.4 Colon length measurement and histopathological score

After the mice were sacrificed, the large intestines were removed and placed flat on white paper. A ruler was placed on the left side of the intestinal segment, and the length from the cecum to the anus was measured, recorded and photographed. Approximately 1 cm of distal colon tissue was removed, placed in 4% paraformaldehyde, fixed at room temperature for 24 h, embedded in paraffin blocks, cut into paraffin sections with a thickness of 4 μm, pasted on glass slides, and stained with hematoxylin and eosin (H&E). A microscope was used to observe the histological changes and obtain the histopathological score (33, 34). The detailed criteria for determining the severity of colonic tissue damage were as follows: degree of inflammatory cell infiltration, none = 0, slight = 1, moderate = 2, severe = 3; percentage of damaged area, no tissue damage = 0, 1–25% = 1, 26–50% = 2, 51–75% = 3, and 76–100% = 4; extent of tissue damage, no damage = 0, limited to mucous membranes = 1, mucous and submucous = 2, beyond submucous = 3; and degree of crypt damage, no damage = 0, basal 1/3 damaged = 1, basal 2/3 damaged = 2, only surface epithelium intact = 3, and loss of entire crypt and epithelium = 4.

### 2.5 Intestinal permeability

Intestinal permeability was assessed using the fluorescein isothiocyanate (FITC)-dextran tracer method. After a 8-h fast with free access to water, mice received an oral gavage of FITC-dextran (4 kDa FD4; Sigma-Aldrich) at a dose of 600 mg/kg. Four hours later, blood was collected via cardiac puncture under anesthesia. Serum was separated by centrifugation, and fluorescence intensity was measured (Ex/Em = 490/520 nm). The serum FITC-dextran concentration was determined against a standard curve prepared from the same stock solution.

### 2.6 Cell culture and drug treatment

Caco-2 cells (ATCC, MD, USA) were cultured in high-glucose DMEM (Gibco, NY, United States) containing 10% fetal bovine serum (FBS, HyClone, UT, United States), 1% penicillin–streptomycin, and 1% non-essential amino acids (Gibco). Cells were incubated at 37 °C in a humidity atmosphere with 5% CO_2_ and 21% O_2_. Subculture was performed every 2–4 days at a split ratio of 1:3 when cells reached approximately 80% confluence, with the culture medium replenished every 48 h. For experiments, cells were seeded into 6-well plates at a density of 1 × 10^6^ cells per well. Following attachment, cells were pretreated for 5 h with EE at the indicated concentrations (25, 50, or 100 μg/mL). Then, 10 μg/mL Lipopolysaccharide (35, 36) (LPS, Sigma-Aldrich, MO, United States) or TNF-α/IFN-γ (10 ng/mL each) (37) was added to the 6-well plate, and the cells were cultured in the incubator continued for 24 h.

### 2.7 Cell viability assay

Caco-2 cells were seeded into 96-well plates at a density of 1 × 10^4^ cells per well and allowed to adhere for 24 h under standard culture conditions (37 °C, 5% CO₂). Subsequently, the cells were exposed to various concentrations of EE (25, 50, and 100 μg/mL) for 24, 48, or 72 h. Cell viability was assessed using the CCK-8 assay kit (Dojindo Laboratories, Kumamoto, Japan). Briefly, 10 μL of CCK-8 reagent was added to each well, followed by incubation at 37 °C for 2 h. Absorbance was measured at 450 nm using a microplate reader (Molecular Devices, MD, United States).

### 2.8 Transepithelial electrical resistance (TEER)

Caco-2 cells were seeded on collagen-coated Transwell inserts (0.4 μm pore, 12 mm diameter, effective membrane area 1.12 cm^2^) and cultured for 21 days to form confluent monolayers. Only inserts with a baseline TEER >500 Ω·cm^2^ were used. TEER was measured at 24 h post-treatment using an epithelial voltohmmeter (EVOM, World Precision Instruments). Net resistance (Ω·cm^2^) was calculated as (R_total − R_blank) × A (*A* = 1.12 cm^2^) and normalized to untreated controls. For each independent experiment, three technical replicates (inserts) were used per group. Three independent biological replicates (*n* = 3) were performed (38).

### 2.9 Western blotting

Western blot analysis was performed following standard procedures. Briefly, colon tissues and Caco-2 cells were lysed in RIPA buffer (Vazyme, Nanjing, China) to extract total protein. Equal amounts of protein lysates were resolved by 12% SDS-PAGE and subsequently transferred to PVDF membranes (Millipore, Darmstadt, Germany). After blocking with a rapid blocking buffer for 15 min at room temperature, the membranes were incubated overnight at 4 °C with the following primary antibodies diluted in blocking buffer: ZO-1 (Servicebio; 1:500), Occludin (Proteintech, Wuhan, China; 1:2000), TNF-*α* (Abcam, CA, United Kingdom; 1:1000), IL-6 (Abcam; 1:1000), IL-1β (Abcam; 1:1000), p-p65 (Beyotime; 1:1000), AKT (Invitrogen; 1:1000), p-AKT (Abcam; 1:1000), PI3K (Abcam; 1:1000), p-PI3K (Invitrogen; 1:1000), and GAPDH (Abcam; 1:5000). Following three washes with TBST, membranes were incubated for 1.5 h at room temperature with HRP-conjugated secondary antibodies (FDbio; 1:40000). Protein bands were detected using an enhanced chemiluminescence (ECL) substrate (FDbio) and quantified with ImageJ software.

### 2.10 Enzyme-linked immunosorbent assay (ELISA)

Serum concentrations of TNF-*α*, IL-1β, and IL-6 were measured using commercial ELISA kits (Elabscience Biotechnology, Wuhan, China). Blood samples were collected from mice at the time of sacrifice, allowed to clot at room temperature for 30 min, and centrifuged at 3,000 × g for 15 min at 4 °C. The resulting serum was stored at −80 °C until analysis. For the assay, 100 μL of standard or diluted serum sample was added to pre-coated wells and incubated for 90 min at 37 °C. After washing, biotinylated detection antibody was added and incubated for 60 min, followed by horseradish peroxidase-conjugated streptavidin for 30 min. Tetramethylbenzidine substrate was added, and the reaction was terminated with stop solution. Absorbance was read at 450 nm using a microplate reader (Molecular Devices M3, United States). Cytokine concentrations were calculated from standard curves generated with known concentrations of recombinant cytokines.

### 2.11 Immunohistochemical (IHC) assay

Colonic tissue sections underwent standard deparaffinization in xylene followed by rehydration through a graded ethanol series. Endogenous peroxidase activity was quenched by incubating the sections with 3% H₂O₂ for 10 min at room temperature, after which they were rinsed three times with PBST. Nonspecific binding was blocked with 5% BSA for 30 min. The sections were then incubated overnight at 4 °C with primary antibodies against ZO-1 (Servicebio, 1:200) and Occludin (Proteintech, 1:400). Following three PBST washes, the sections were exposed to HRP-conjugated secondary antibodies (Servicebio, 1:200) for 1 h at room temperature. Antibody binding was visualized using DAB substrate (Servicebio).

### 2.12 Immunofluorescence (IF) assay

Caco-2 cells (1 × 10^4^ cells/well) were evenly seeded in a 24-well plate covered with round coverslips, cultured for 24 h, and pretreated with different concentrations of EE (25, 50, and 100 μg/mL) for 5 h. Then, 10 μg/mL LPS was added, and the cells were cultured for 24 h. After aspirating and discarding the culture medium, 4% paraformaldehyde was added to the round coverslips to fix the cells for 20 min. The cells were then washed with PBS three times, blocked with 5% BSA for 30 min, and incubated with rabbit anti-ZO-1 (Servicebio; 1:200) and rabbit anti-Occludin (Proteintech; 1:200) overnight at 4 °C. After washing with PBST three times, the round coverslips were incubated with Alexa Fluor 488 (Abcam; 1:2000) and Cy3 (Abcam; 1:2000) at room temperature in the dark for 1.5 h. After washing with PBST three times, DAPI (Abcam; 1:2000) was used to stain the nuclei under dark conditions. Finally, the round coverslips were examined using an FV3000 confocal laser scanning microscope for fluorescence intensity examination and imaging.

### 2.13 Quantitative real-time PCR (qRT-PCR)

Following the manufacturer’s protocols, total RNA was purified from cell samples using the FastPure Cell/Tissue Total RNA Isolation Kit (Vazyme, China). After determining RNA concentration and purity, 1 μg of total RNA from each sample was reverse-transcribed into cDNA with the HiScript RT SuperMix kit (Vazyme). Quantitative real-time PCR assays were run in triplicate on a CFX96 system (Bio-Rad, United States) using ChamQ Universal SYBR qPCR Master Mix (Vazyme). The relative quantification of mRNA expression was performed using the 2^−ΔΔCt^ method, with GAPDH serving as the internal control. Primer sequences are detailed in Table 1 (28).

**Table 1:** The primer sequences of qRT-PCR.

Gene	Primer sequence (5′-3′)	
	Forward	Reverse
*TNF-α*	CCTCTCTCTAATCAGCCCTCTG	GAGGACCTGGGAGTAGATGAG
*IL-1β*	AGCTACGAATCTCCGACCAC	CGTTATCCCATGTGTCGAAGAA
*IL-6*	ACTCACCTCTTCAGAACGAATTG	CCATCTTTGGAAGGTTCAGGTTG
*GAPDH*	GGAGCGAGATCCCTCCAAAAT	GGCTGTTGTCATACTTCTCATGG

### 2.14 Network pharmacology analysis

To elucidate the potential mechanisms of EE, a network pharmacology analysis approach was employed. Chemical components of earthworm were retrieved from published LC–MS/MS data and traditional Chinese medicine databases, including TCMBANK^1^ and TCM Data-base@Taiwan. These candidate compounds were screened using the following criteria: (1) compliance with at least two out of five drug-likeness filters (Lipinski, Ghose, Veber, Egan, Muegge); (2) a bioavailability score ≥ 0.55 predicted by SwissADME;^2^ and (3) availability of canonical SMILES notations. Potential protein targets of the screened compounds were predicted using the SwissTargetPrediction platform.^3^ Disease-related targets for UC were obtained from the GeneCards database, and those with a relevance score ≤ 1 were excluded.

The overlapping targets between EE components and UC were identified using an online Venn diagram tool^4^. The resulting intersection targets were imported into the STRING database^5^ to construct a protein–protein interaction (PPI) network. This network was further analyzed and visualized using Cytoscape software (version 3.7.0). Gene Ontology (GO) and Kyoto Encyclopedia of Genes and Genomes (KEGG) pathway enrichment analyses for the intersection targets were performed using the DAVID bioinformatics database^6^. Enrichment results were visualized as bubble plots using an online platform^7^.

### 2.15 Data analysis

Statistical analyses were performed using GraphPad Prism version 9.5 (GraphPad Software, United States). Normality of the data was verified with the Shapiro–Wilk test, and homogeneity of variances was assessed using Levene’s test. Depending on data distribution and experimental design, between-group comparisons were conducted using Student’s *t*-test, one-way ANOVA, or two-way ANOVA, as appropriate. Data are presented as mean ± standard error of the mean (SEM) from at least three independent replicates. A *p*-value below 0.05 was considered statistically significant. The following symbols denote significance levels in figures: *p < 0.05, **p < 0.01, ***p < 0.001, ****p < 0.0001; “ns” indicates not significant.

## 3 Results

### 3.1 Quality control of EE and its effect on DSS-induced colitis

The EE, primarily composed of proteins and peptides (21), was first characterized for its quality consistency and endotoxin levels. For quality control purposes, HPLC analysis was performed to establish the chemical fingerprint of EE. As shown in Supplementary Figures S1A,B, four characteristic peaks were identified by comparison with reference standards as hypoxanthine, uridine, inosine, and guanosine. Fingerprint analysis of eight independently prepared batches demonstrated good batch-to-batch consistency (>0.99, Supplementary Figure S1C). The endotoxin level in the treated EE was measured at 0.109 EU/mL, indicating a relatively low level (Supplementary Table S1).

**Figure 1:**
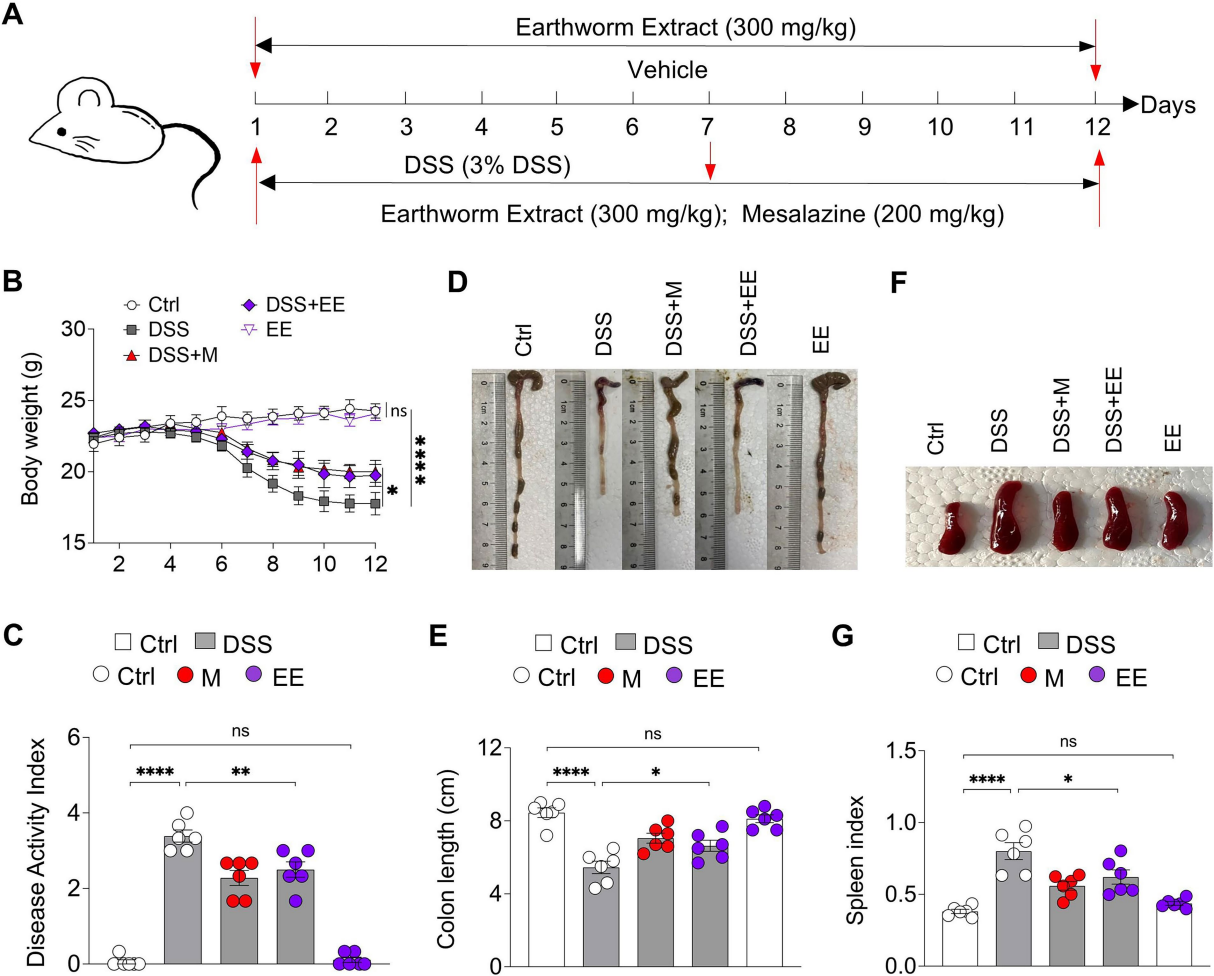
EE improves disease severity in DSS-induced colitis. **(A)** Schematic diagram of the experimental timeline for DSS-induced colitis and treatment with EE or mesalazine. **(B)** Daily changes in body weight among the experimental groups. **(C)** DAI score. **(D)** Representative images of colons. **(E)** Quantification of colon length. **(F)** Representative photographs of spleens. **(G)** Corresponding spleen weight. Data are presented as mean ± SEM. *p < 0.05, **p < 0.01, ****p < 0.0001; ns, not significant.

Having established the quality and safety profile of EE, we next evaluated its therapeutic effect in a murine model of UC. A murine model of UC was successfully established by administering 3% DSS in drinking water ad libitum to C57BL/6 mice for 7 days (Figure 1A). Disease progression was monitored daily by calculating a disease activity index (DAI) based on body weight loss, stool consistency, and fecal occult blood. DSS-treated mice exhibited progressive weight loss from day 5 onward, which became statistically significant by day 6 (Figure 1B). The therapeutic potential of earthworm extract (EE) was evidenced by its significant attenuation of this DSS-induced weight loss, an effect comparable to that of the positive control drug mesalazine (Figure 1B). Furthermore, the model group manifested severe colitis phenotypes, including a significantly elevated DAI score, marked colon shortening, and notable splenomegaly compared to the normal control and EE-only groups (Figures 1C–G). Importantly, EE treatment significantly mitigated all these pathological changes (Figures 1C–G). The absence of significant adverse effects in the EE-only group, as reflected by normal body weight, DAI, colon length, and spleen size (Figures 1B–G), indicates good short-term tolerability of the extract in mice.

### 3.2 EE alleviates DSS-induced colonic tissue damage without causing short-term toxicity

**Figure 2:**
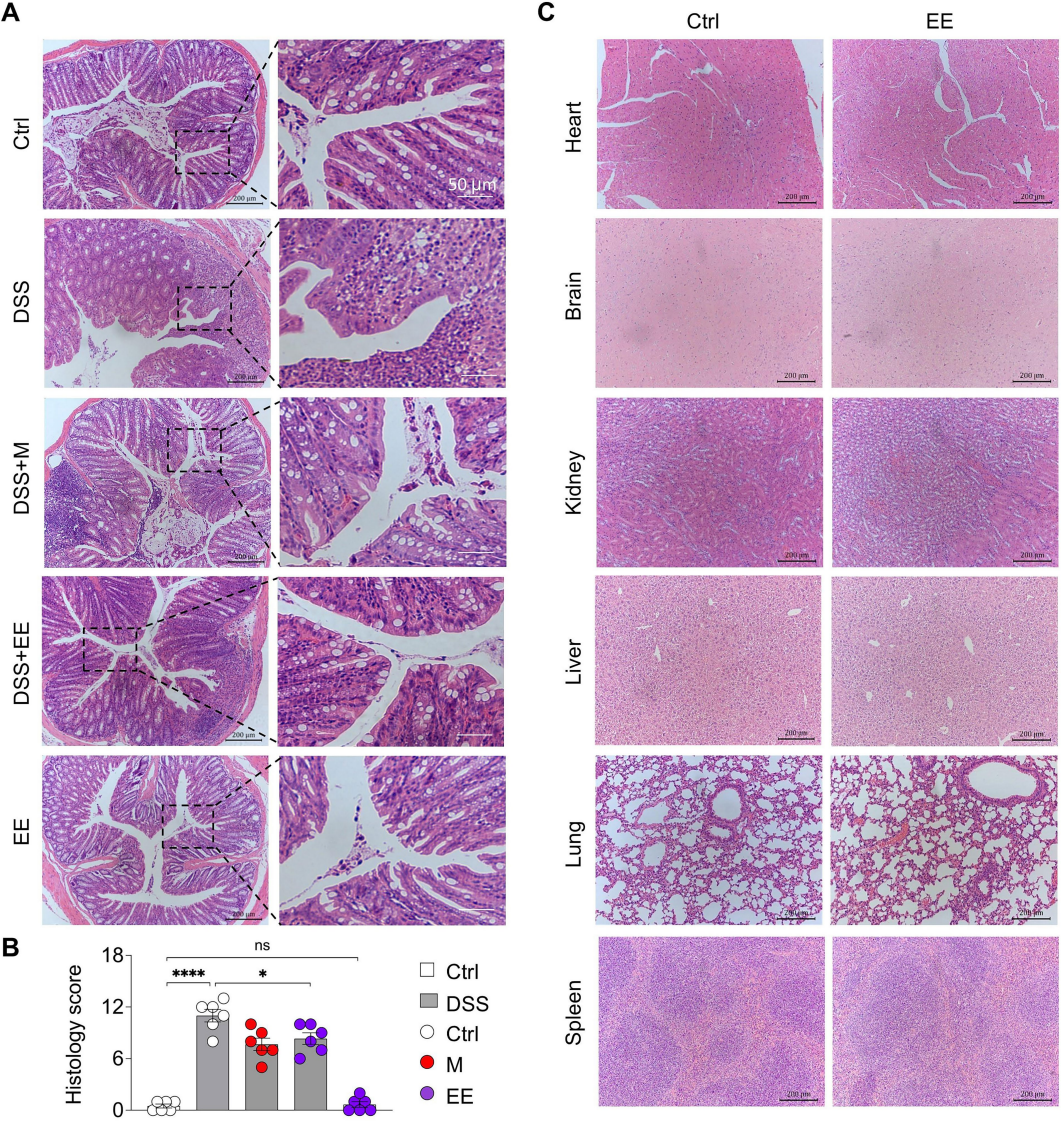
EE alleviates DSS-induced colonic tissue damage without causing short-term toxicity. **(A)** Representative images of colon sections stained with H&E. Scale bar = 200 μm. **(B)** Histology score (HS). The histology score reflects the degree of colonic damage. **(C)** Representative photographs of heart, brain, kidney, liver, lung, and spleen sections from H&E staining. Scale bar = 200 μm. Data are presented as mean ± SEM. ^*^p < 0.05, ****p < 0.0001; ns, not significant.

Hematoxylin and eosin (H&E) staining was performed to evaluate the histopathological alterations in colonic tissues. In contrast to the intact epithelium, well-defined crypts, and absence of significant inflammation observed in normal controls, DSS-treated mice exhibited severe mucosal damage characterized by epithelial erosion, crypt distortion, and extensive inflammatory cell infiltration (Figure 2A). Consequently, this group displayed a significantly elevated histopathological score (Figure 2B). Treatment with EE conferred remarkable protection against these structural alterations. Colonic tissues from the EE-treated group maintained substantially preserved epithelial integrity, with clearly discernible crypts, alongside a marked reduction in leukocyte infiltration (Figure 2A), which corresponded to a significantly lower histopathological score (Figure 2B). The protective efficacy of EE was comparable to that of the standard drug mesalazine.

Furthermore, to assess systemic safety, healthy mice were administered EE alone for 12 consecutive days. No mortality or signs of acute short-term toxicity – such as alterations in body weight, fecal consistency, fur condition, or spontaneous behavior – were observed throughout the treatment period. Gross necropsy revealed no abnormalities in major organs. Histopathological examination of H&E-stained sections from the heart, liver, spleen, lungs, kidneys, and brain confirmed the absence of treatment-related lesions, inflammation, or structural alterations (Figure 2C). Collectively, these results demonstrate that EE not only ameliorates DSS-induced colonic damage but also exhibits excellent biocompatibility and a favorable safety profile *in vivo*.

### 3.3 EE ameliorates the NF-κB-mediated inflammatory response

**Figure 3:**
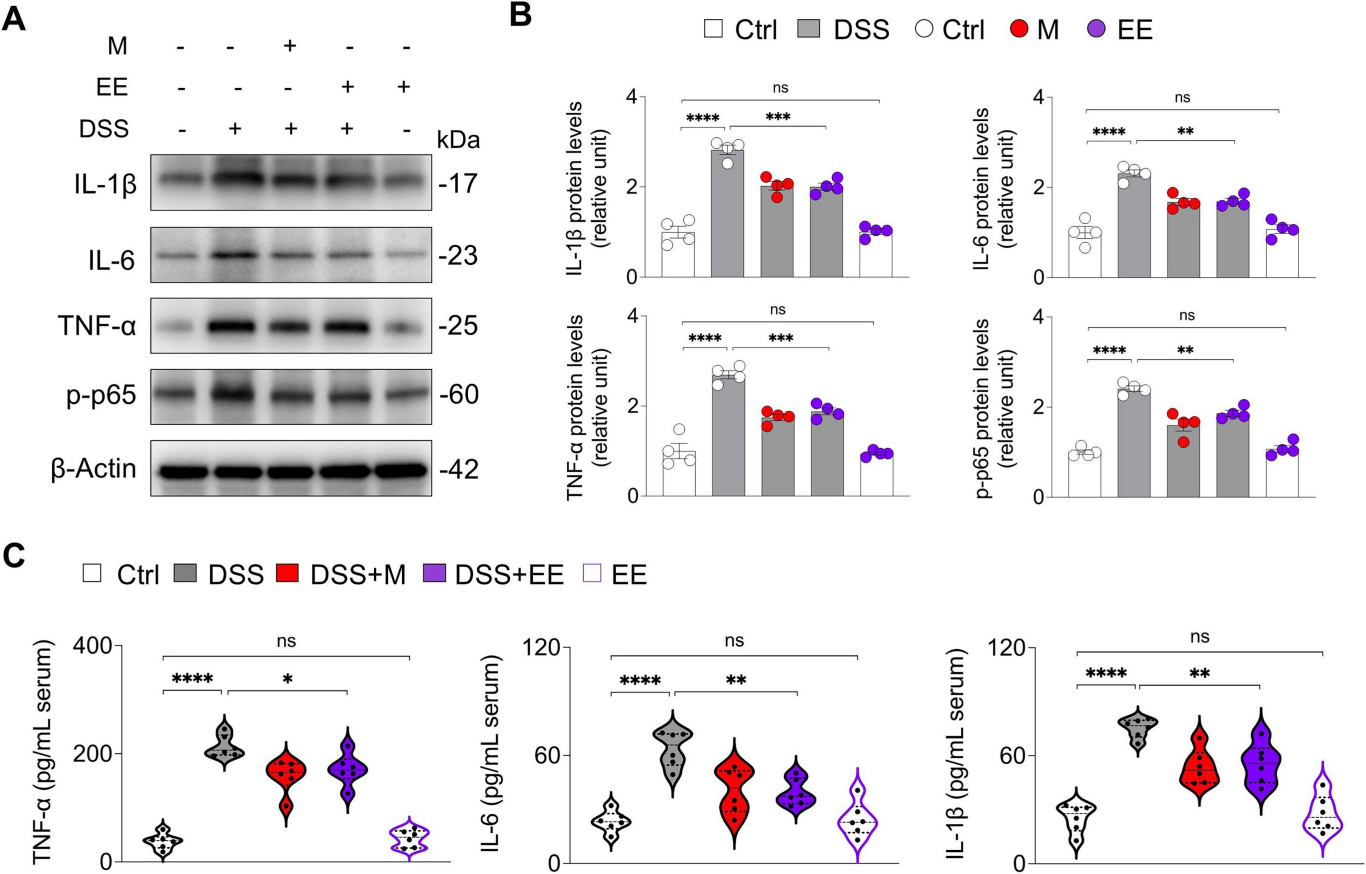
EE ameliorates the NF-κB-mediated inflammatory response. **(A)** Western blot analysis of pro-inflammatory cytokines TNF-*α*, IL-6, and IL-1β in colon tissues from the indicated groups. **(B)** Densitometric quantification of the blots shown in **(A)**. **(C)** Serum levels of TNF-α, IL-6, and IL-1β measured by ELISA. Data are presented as mean ± SEM. *p < 0.05, **p < 0.01, ***p < 0.001, ****p < 0.0001; ns, not significant.

The UC is characterized by the production of various inflammatory factors, such as TNF-α, IL-1β, and IL-6, which play important roles in the occurrence and development of UC (14). To investigate the anti-inflammatory mechanism of EE, we analyzed key inflammatory mediators associated with UC pathogenesis. The expression levels of pro-inflammatory cytokines (TNF-α, IL-1β, and IL-6,) in colon tissue and serum were quantified by Western blotting and ELISA. As expected, DSS challenge markedly upregulated the production of these cytokines compared to the normal control group, EE intervention significantly suppressed these DSS-induced elevations in TNF-α, IL-6, and IL-1β (Figures 3A–C). Furthermore, to probe the underlying signaling pathway, we examined the activation of NF-κB, a master regulator of inflammation, by assessing the phosphorylation of its key subunit p65. Consistent with the cytokine profile, the level of phospho-p65 (p-p65) was substantially increased in the colon tissue of DSS-treated mice, and this activation was potently reversed by EE treatment (Figures 3A,B). Importantly, EE administration alone to healthy mice did not alter the basal levels of these inflammatory markers or p-p65, indicating its action is specific to the inflammatory context and exhibits a favorable safety profile (Figures 3A–C). Collectively, these data demonstrate that EE alleviates experimental colitis by inhibiting the NF-κB signaling pathway and downstream pro-inflammatory cytokine production.

### 3.4 EE restores intestinal barrier integrity and upregulates TJ protein expression

**Figure 4:**
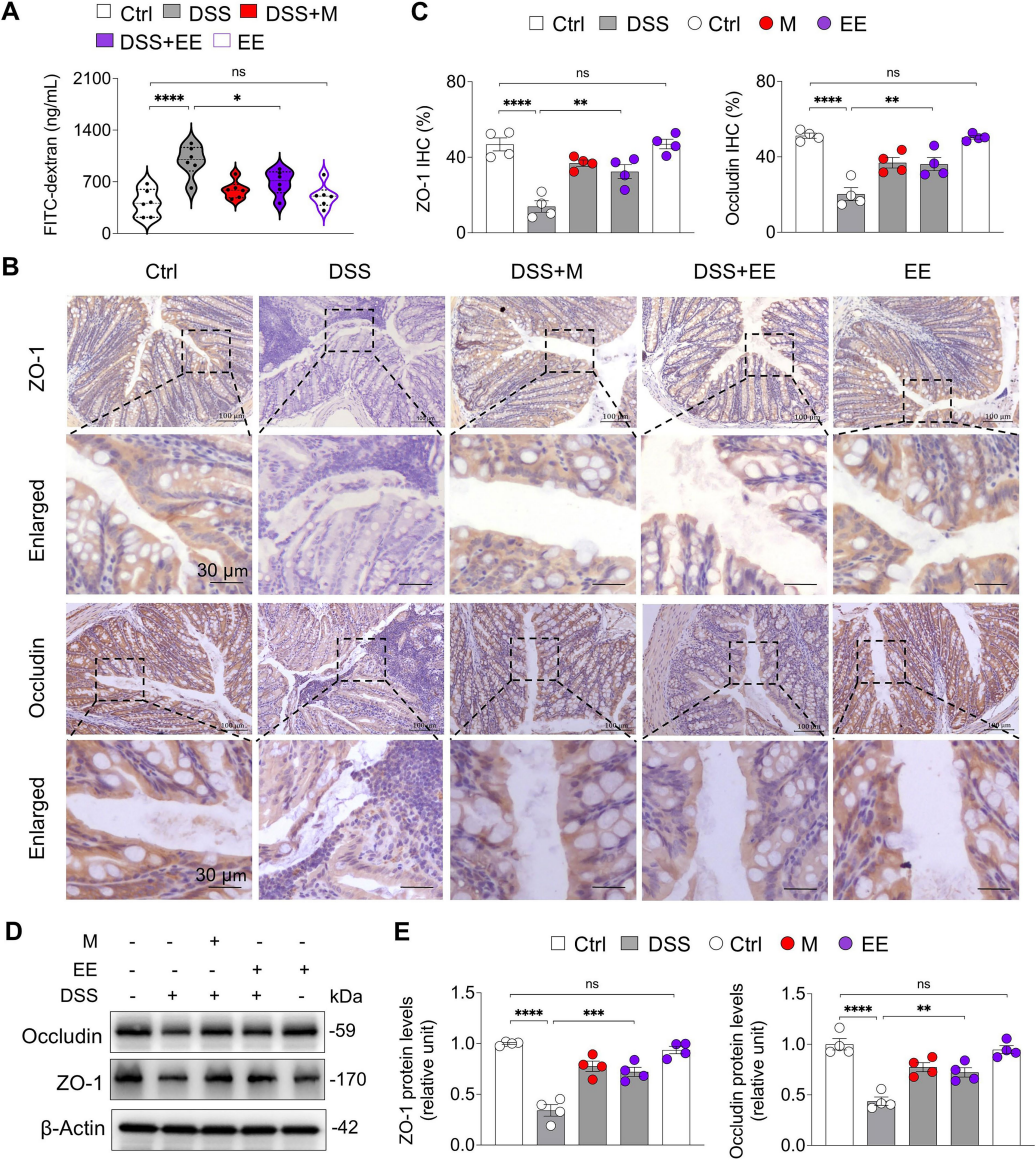
EE restores intestinal barrier integrity and upregulates TJ protein expression. **(A)** Intestinal permeability was assessed using the FITC-dextran tracer assay. **(B)** Representative images of immunohistochemical staining for ZO-1 and Occludin are displayed. Scale bar = 100 μm. **(C)** Quantification of immunofluorescence staining intensity for ZO-1 and Occludin. **(D)** Western blot analysis of TJ proteins (ZO-1 and Occludin) in colon tissues. **(E)** Densitometric analysis of protein levels shown in **(D)**. Data are presented as mean ± SEM. *p < 0.05, **p < 0.01, ***p < 0.001, ****p < 0.0001; ns, not significant.

The integrity of the intestinal epithelial barrier, critically maintained by TJ proteins such as ZO-1 and Occludin, is profoundly compromised in UC, leading to increased permeability (39). Functional assessment using the FITC-dextran flux assay confirmed that DSS challenge significantly elevated intestinal permeability, which was markedly attenuated by EE treatment (Figure 4A). To elucidate the structural basis of this functional improvement, we examined the expression and localization of key TJ proteins. Immunohistochemical (IHC) evaluation revealed that DSS induction led to a marked diminution and disorganization of ZO-1 and Occludin staining at the colonic epithelial cell. Conversely, EE administration effectively preserved the characteristic continuous, linear distribution of these proteins along the crypt and significantly augmented their staining intensity relative to the DSS model group (Figures 4B,C). Consistently, Western blot analysis of colonic tissues demonstrated that the DSS-induced downregulation of ZO-1 and Occludin protein levels was significantly reversed by EE administration (Figures 4D,E). Collectively, these multi-faceted data indicate that EE ameliorates intestinal barrier dysfunction in experimental colitis by enhancing the expression and proper localization of critical TJ proteins, thereby reducing pathological permeability.

### 3.5 EE preserves intestinal barrier integrity in LPS-challenged Caco-2 cells

**Figure 5:**
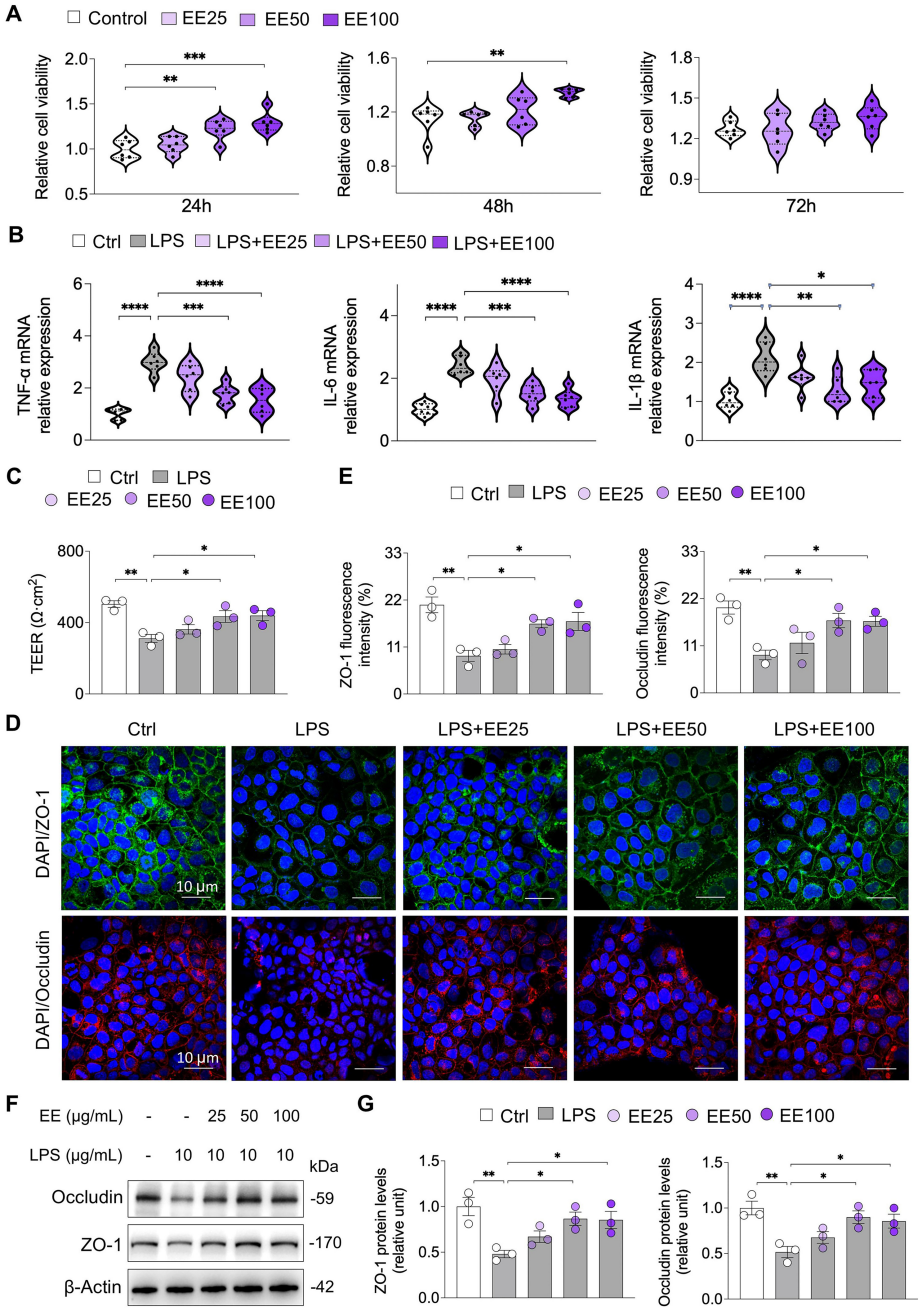
EE protects intestinal epithelial cells against LPS-induced barrier disruption via anti-inflammatory and junction-enhancing. **(A)** Cell viability of Caco-2 cells treated with EE (25, 50, 100 μg/mL) for 24, 48, and 72 h, as assessed by the CCK-8 assay. **(B)** Relative mRNA expression of pro-inflammatory cytokines (TNF-α, IL-6, and IL-1β) in Caco-2 cells following LPS stimulation with or without EE pretreatment. **(C)** Transepithelial electrical resistance (TEER) of Caco-2 monolayers after LPS challenge in the presence or absence of EE. **(D,E)** Immunofluorescence staining showing the distribution of ZO-1 (green) and Occludin (red) in Caco-2 monolayers, with nuclei counterstained by DAPI (blue), and corresponding quantitative analysis of fluorescence intensity. **(F,G)** Western blot analysis of ZO-1 and Occludin protein expression **(F)** and densitometric quantification of the immunoblots **(G)**. Data are presented as mean ± SEM. *p < 0.05, **p < 0.01, ***p < 0.001, ****p < 0.0001.

Prior to functional experiments, the biocompatibility of EE was assessed in Caco-2 intestinal epithelial cells. CCK-8 assays showed that exposure to EE alone did not compromise cell viability; instead, a concentration-dependent increase in viability was observed relative to untreated cells (Figure 5A). Furthermore, to evaluate whether EE alone induced inflammatory responses, Caco-2 cells were treated with EE. The qPCR assay showed that EE alone did not induce the expression of TNF-α, IL-1β, or IL-6 compared to the negative control (Supplementary Figure S2), indicating that the residual endotoxin in EE did not trigger a detectable inflammatory response in our cell culture system. To further investigate the direct protective effects of EE on the intestinal epithelial barrier, we employed an *in vitro* model of LPS-challenged Caco-2 cells. Firstly, EE dose-dependently suppressed the LPS-induced inflammatory response, as evidenced by a significant reduction in the mRNA expression levels of key pro-inflammatory cytokines (TNF-α, IL-6, and IL-1β) (Figure 5B). Concurrently, functional analysis demonstrated that EE markedly ameliorated the LPS-induced impairment of barrier integrity, with a significant increase in transepithelial electrical resistance (TEER) across the Caco-2 monolayers (Figure 5C). Immunofluorescence staining revealed that LPS exposure led to a discontinuous distribution and diminished fluorescence intensity of ZO-1 and Occludin at the cell borders, indicating TJ disruption (Figures 5D,E). EE treatment effectively reversed this damage, restoring coherent membranous localization and enhancing the fluorescence signal of both proteins (Figures 5D,E). Consistent with these findings, Western blot analysis confirmed that the LPS-induced downregulation of ZO-1 and Occludin protein expression was significantly reversed by EE (Figures 5F,G). Collectively, these in vitro data demonstrate that EE protects against LPS-induced barrier dysfunction by a dual mechanism: attenuating the upstream inflammatory cascade and directly upregulating the expression and promoting the proper assembly of key TJ proteins, thereby restoring epithelial integrity.

### 3.6 Network pharmacology analysis of the mechanisms of EE against DSS-induced colitis

**Figure 6:**
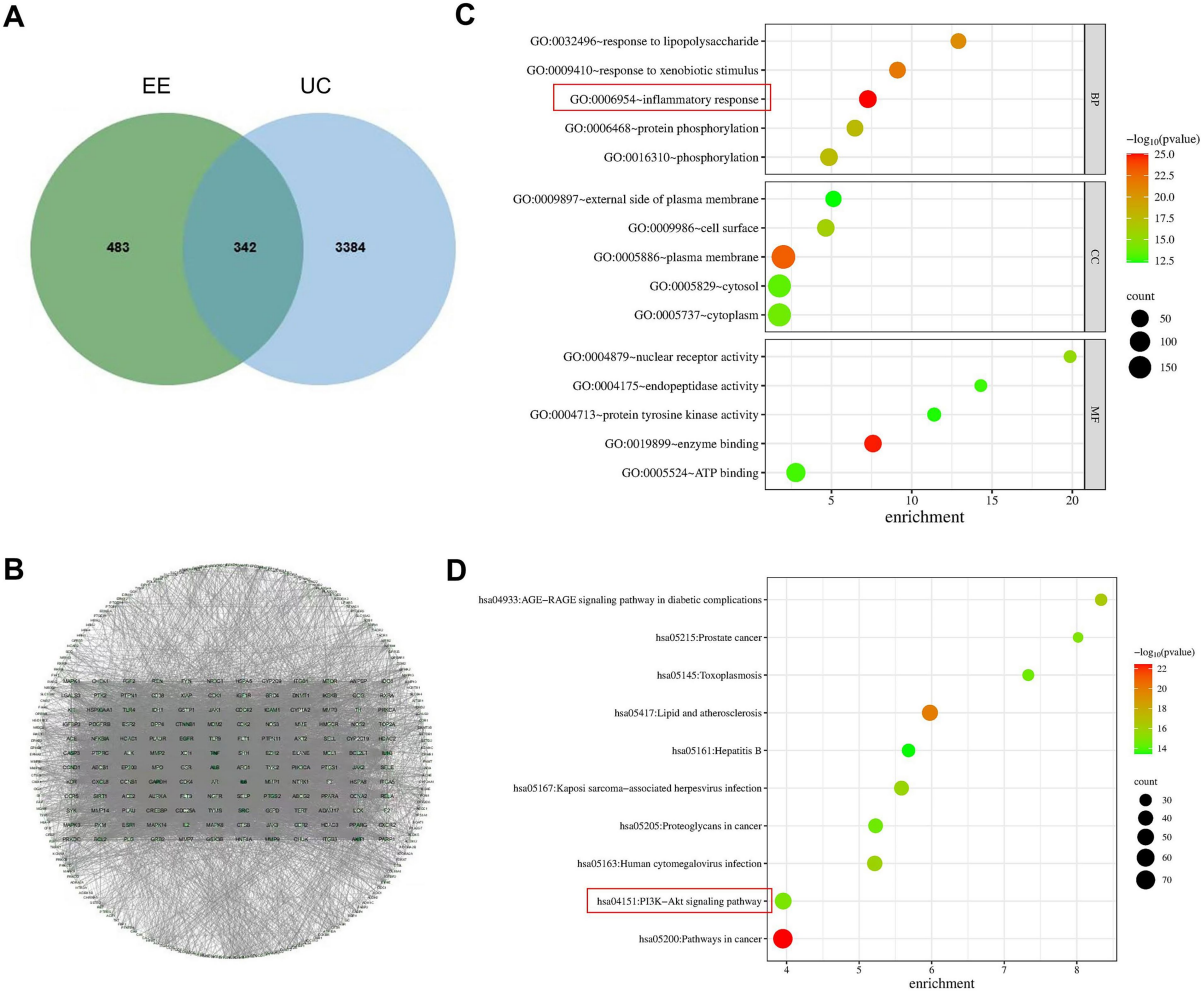
Network pharmacology analysis of the mechanisms of EE against DSS-induced colitis. **(A)** Venn diagram showing the overlap between predicted targets of EE bioactive compounds and known UC-related targets, yielding 342 intersecting targets. **(B)** Protein–protein interaction (PPI) network of the 342 intersecting targets. Key hub nodes are highlighted, with node size and color intensity representing the degree of connectivity. **(C)** Bubble plot of the top enriched GO terms for the intersecting targets, categorized into biological process (BP), cellular component (CC), and molecular function (MF). **(D)** Bubble plot of the top enriched KEGG pathways for the intersecting targets. The size of the bubble indicates the number of genes enriched in the pathway, and the color represents the statistical significance [−log10(*p*-value)].

To ensure a comprehensive analysis, 273 bioactive compounds identified from EE were included (Supplementary material). After removing duplicates, a total of 825 compound-related targets and 3,726 UC-associated targets were retrieved, yielding 342 overlapping targets as potential key mediators (Figure 6A). A protein–protein interaction (PPI) network was constructed using the STRING database and visualized with Cytoscape 3.7.0. Network topology analysis identified several highly connected hub genes, such as AKT1, IL-6, TNF, SRC, and EGFR (Figure 6B), suggesting their central role in the mechanism of EE against UC. Functional enrichment of these intersection targets was subsequently performed. Gene Ontology (GO) analysis revealed that biological processes (BP) were prominently associated with ‘response to lipopolysaccharide’ and ‘inflammatory response’ (Figure 6C). Kyoto Encyclopedia of Genes and Genomes (KEGG) pathway enrichment highlighted significant involvement in several signaling cascades, such as ‘Pathways in cancer’, ‘PI3K-AKT signaling pathway’, and ‘Human cytomegalovirus infection’ (Figure 6D). These enrichment results strongly indicate that the therapeutic effect of EE in UC may be closely linked to the modulation of the PI3K-AKT and TNF-mediated inflammatory axes.

### 3.7 EE inhibits the activation of the PI3K/AKT pathway *in vivo* and *in vitro*

**Figure 7:**
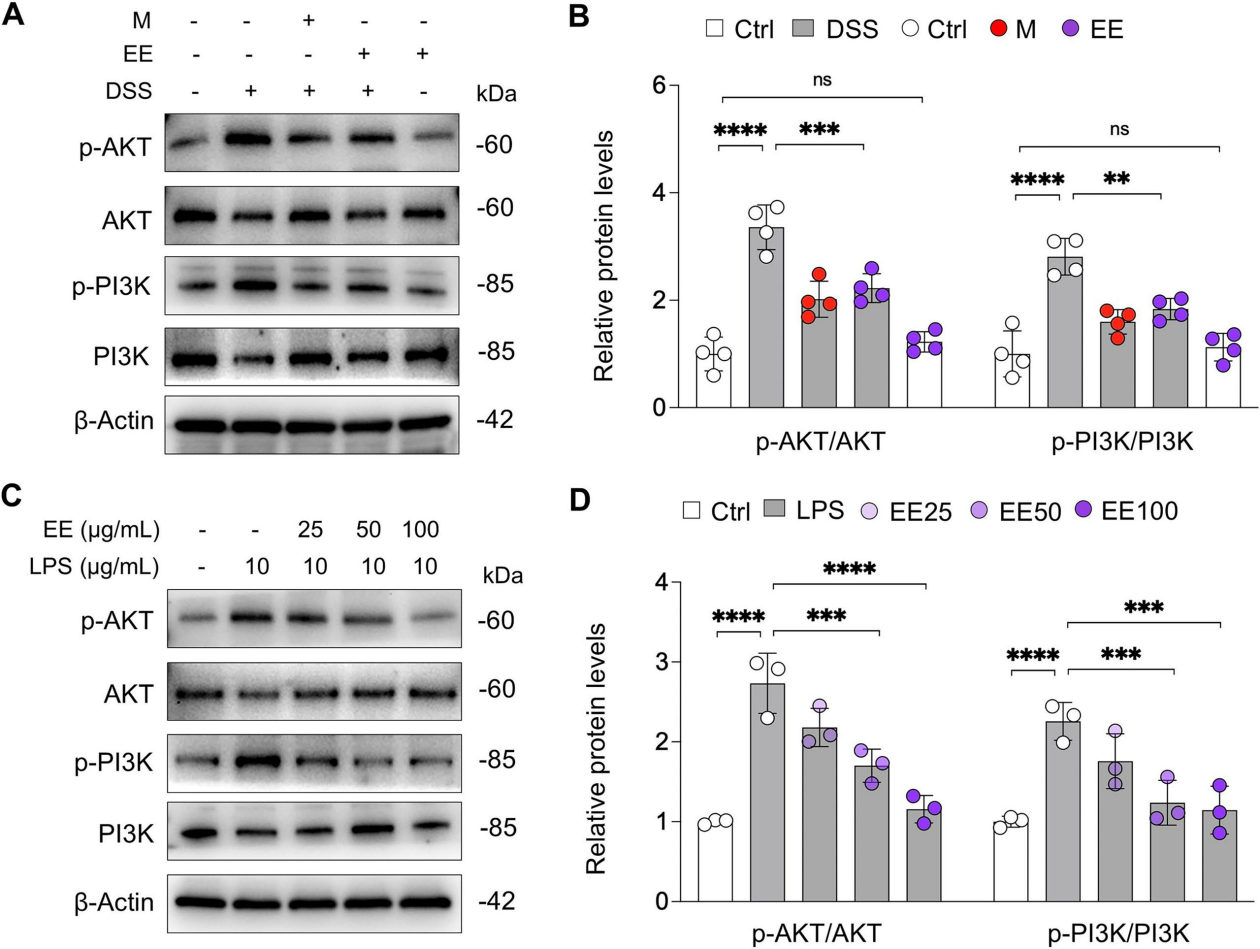
EE inhibits the activation of the PI3K/AKT pathway *in vivo* and *in vitro*. **(A,B)** Western blot analysis of p-PI3K, PI3K, p-AKT, and AKT protein levels in colon tissues from the indicated groups. Bar graphs show the quantified p-PI3K/PI3K and p-AKT/AKT ratios. **(C,D)** Caco-2 cells were pretreated with the indicated concentrations of EE (25, 50, and 100 μg/mL) for 5 h, followed by LPS stimulation for 24 h. Representative blots and quantified ratios of p-PI3K/PI3K and p-AKT/AKT are shown. Data are presented as mean ± SEM. **p < 0.01, ***p < 0.001, ****p < 0.0001.

Western blot analysis demonstrated a marked activation of the PI3K/AKT pathway in the colon tissues of DSS-treated mice, reflected by significantly elevated ratios of p-PI3K/PI3K and p-AKT/AKT. EE treatment effectively normalized these phosphorylation levels, with an efficacy comparable to that of the reference drug mesalazine (Figures 7A,B). Notably, EE administration alone in healthy control mice did not affect the basal activity of this pathway, indicating that its suppressive effect is context-dependent and does not interfere with physiological signaling. A parallel pattern was observed in vitro. LPS challenge robustly enhanced PI3K and AKT phosphorylation in Caco-2 cells. This activation was dose-dependently counteracted by EE pretreatment (25, 50, and 100 μg/mL), as shown by the significant reduction in both p-PI3K/PI3K and p-AKT/AKT ratios (Figures 7C,D).

### 3.8 Pharmacological activation of PI3K abolishes the therapeutic effects of EE in DSS-induced colitis

**Figure 8:**
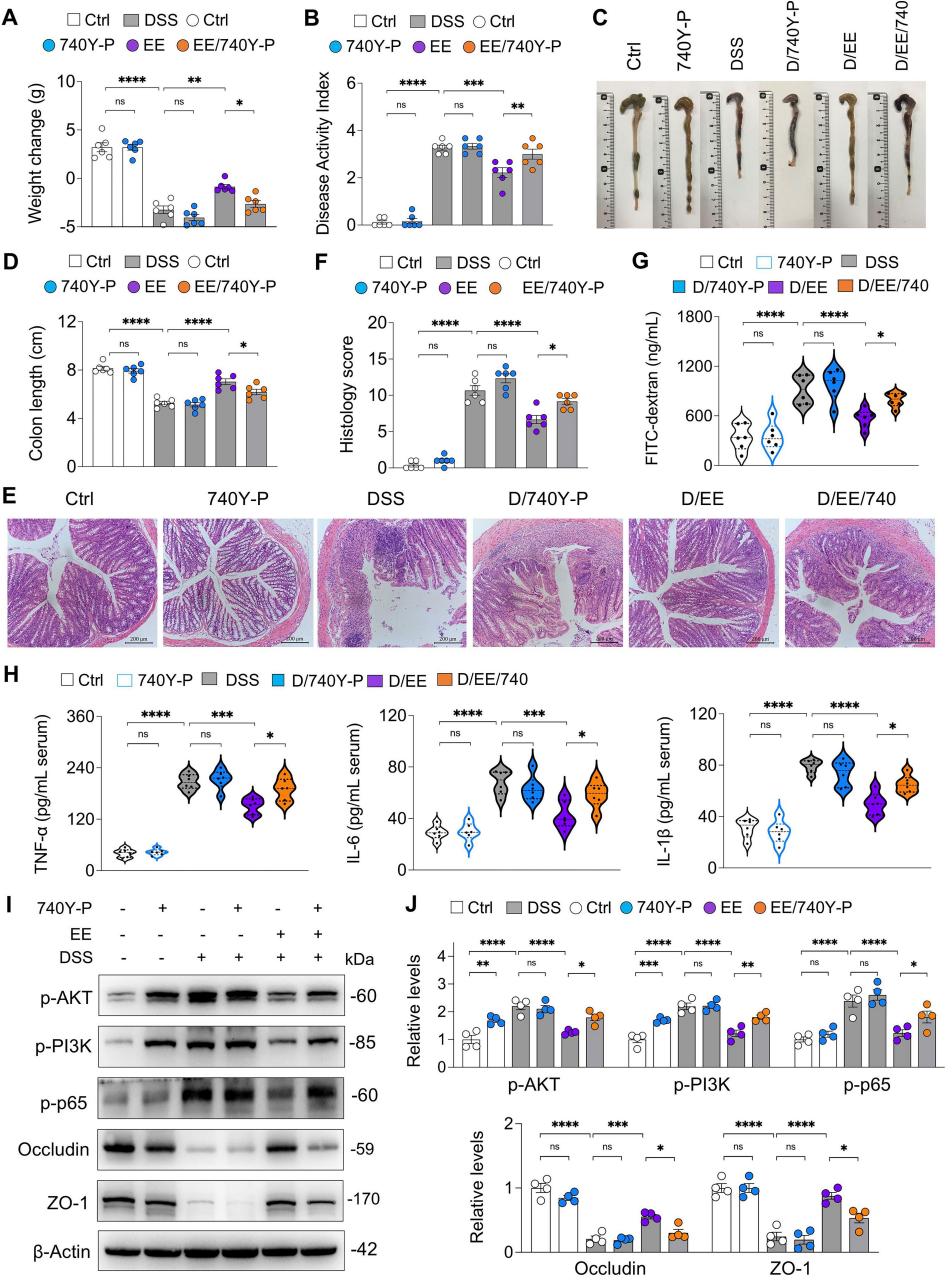
Pharmacological activation of PI3K abolishes the therapeutic effects of EE in DSS-induced colitis. **(A)** Body weight changes across experimental groups. **(B)** DAI score. **(C,D)** Representative colon images **(C)** and quantified colon length **(D)** at the endpoint. **(E,F)** Representative H&E-stained colon sections (**E**; scale bar = 200 μm) and corresponding histopathological scores **(F)**. **(G)** Intestinal permeability assessed by serum FITC-dextran flux. **(H)** Serum levels of pro-inflammatory cytokines (TNF-α, IL-6, and IL-1β) measured by ELISA. **(I,J)** Western blot analysis of p-AKT, p-PI3K, p-p65, Occludin, and ZO-1 protein expression **(I)** and densitometric quantification of the immunoblots **(J)**. Data are presented as mean ± SEM. *p < 0.05, **p < 0.01, ***p < 0.001, ****p < 0.0001; ns, not significant.

To investigate whether the therapeutic effect of EE depends on PI3K/AKT pathway inhibition, we administered the PI3K-specific activator 740Y-P alongside EE in DSS-treated mice. EE administration significantly attenuated DSS-induced body weight loss, disease activity index (DAI), colon shortening, histopathological damage, intestinal permeability, and serum pro-inflammatory cytokines (TNF-α, IL-6, and IL-1β). Co-administration of 740Y-P largely reversed these protective effects (Figures 8A–H). Importantly, administration of 740Y-P alone (Ctrl+740Y-P) did not induce colitis in healthy mice, and the DSS + 740Y-P group showed no significant differences compared with the DSS group, indicating that PI3K activation alone does not exacerbate disease severity under inflammatory conditions (Figures 8A–H).

To confirm the specificity of 740Y-P and its effect on PI3K/AKT signaling, we performed Western blot analysis of colon tissues. As shown in Figure 8I, P-PI3K and p-AKT levels were elevated in the DSS group compared with Ctrl, and EE treatment markedly reduced their expression. Co-administration of 740Y-P with EE partially restored p-PI3K and p-AKT levels. The DSS + 740Y-P group exhibited p-PI3K and p-AKT levels comparable to the DSS group, while the Ctrl+740Y-P group showed elevated levels relative to Ctrl, confirming that 740Y-P acts as a specific activator of this pathway without exacerbating colitis (Figures 8I,J). Additionally, EE reduced p-p65 expression and upregulated ZO-1 and Occludin, effects that were also counteracted by 740Y-P (Figure 8J). The DSS + 740Y-P group showed p-p65, ZO-1, and Occludin levels similar to the DSS group, further supporting that PI3K activation alone does not significantly alter inflammation or barrier integrity (Figures 8I,J). Collectively, these data demonstrate that pharmacological activation of PI3K abolishes the protective effects of EE, providing direct evidence that EE exerts its therapeutic action primarily through inhibition of the PI3K/AKT signaling pathway.

### 3.9 Pharmacological activation of PI3K abolishes the protective effects of EE in TNF-α/IFN-γ-stimulated Caco-2 cells

**Figure 9:**
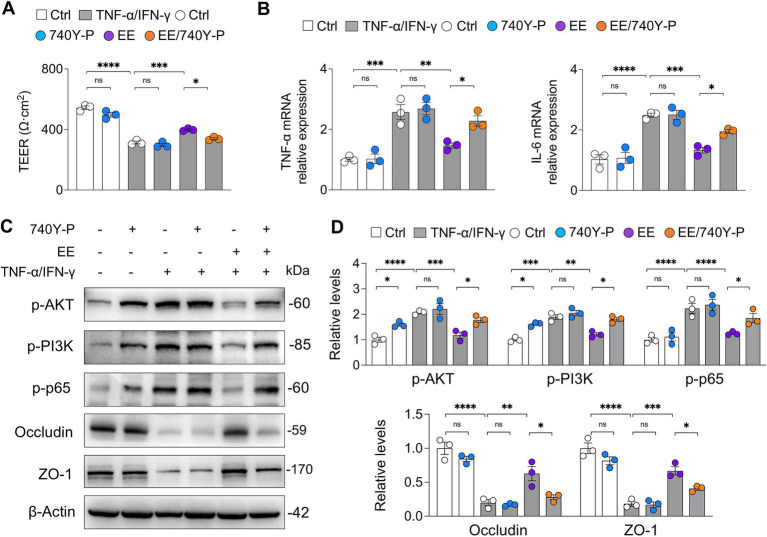
Pharmacological activation of PI3K abolishes the protective effects of EE in TNF-α/IFN-γ-stimulated Caco-2 cells. **(A)** TEER of Caco-2 monolayers after TNF-α/IFN-γ challenge with or without EE (50 μg/mL) in the absence or presence of 740Y-P (10 μm) for 24 h. **(B)** Relative mRNA expression of pro-inflammatory cytokines (TNF-α, IL-6). **(C,D)** Western blot analysis of p-AKT, p-PI3K, p-p65, Occludin, and ZO-1 protein expression **(C)** and densitometric quantification of the immunoblots **(D)**. Data are presented as mean ± SEM. *p < 0.05, **p < 0.01, ***p < 0.001, ****p < 0.0001; ns, not significant.

To further investigate whether the protective effects of EE depend on PI3K/AKT pathway inhibition in intestinal epithelial cells, we used a Caco-2 monolayer model stimulated with TNF-α/IFN-γ. As shown in Figure 9A, TNF-α/IFN-γ stimulation significantly decreased transepithelial electrical resistance (TEER), indicating impaired barrier integrity. EE treatment markedly restored TEER, whereas co-administration of the PI3K activator 740Y-P largely reversed this protective effect. The Ctrl+740Y-P and TNF-α/IFN-γ + 740Y-P groups showed no significant differences compared with the Ctrl and TNF-α/IFN-γ groups, respectively, confirming that 740Y-P alone does not alter barrier function (Figure 9A). Consistent with the TEER results, TNF-α/IFN-γ stimulation upregulated the mRNA expression of TNF-α and IL-6, which were reduced by EE treatment. Co-administration of 740Y-P partially reversed these effects (Figure 9B). Western blot analysis revealed that EE reduced p-PI3K, p-AKT, and p-p65 expression induced by TNF-α/IFN-γ, effects that were counteracted by 740Y-P (Figures 9C,D). Additionally, EE upregulated ZO-1 and Occludin expression, which were also reversed by 740Y-P (Figures 9C,D). The TNF-α/IFN-γ + 740Y-P group showed levels comparable to the TNF-α/IFN-γ group, further supporting the specificity of 740Y-P. Collectively, these results demonstrate that pharmacological activation of PI3K abolishes the protective effects of EE, confirming that EE exerts its protective action in intestinal epithelial cells primarily through inhibition of the PI3K/AKT signaling pathway.

### 3.10 Characterization of the bioactive components in EE

**Figure 10:**
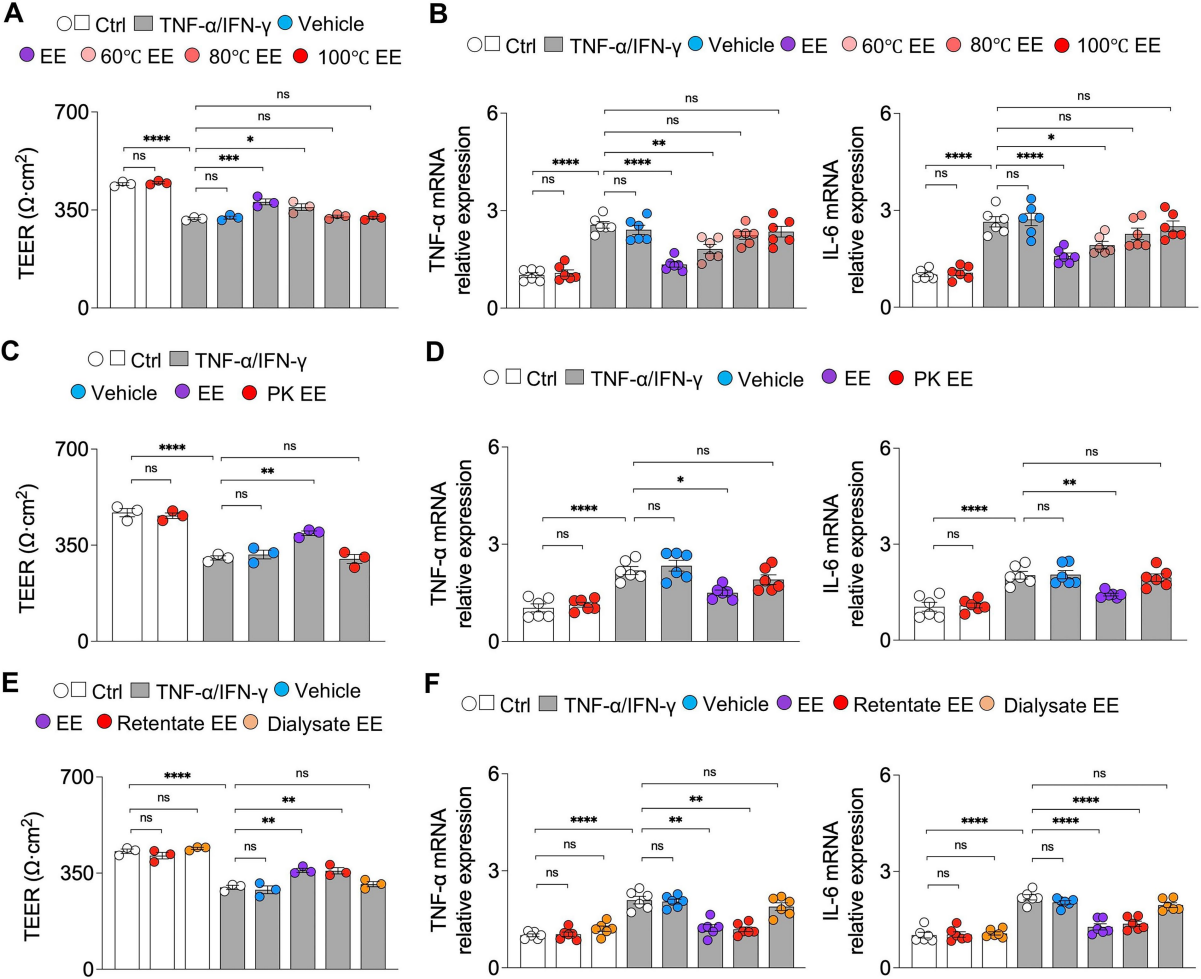
Characterization of the bioactive components in EE. **(A,B)** TEER of Caco-2 monolayers **(A)** and relative mRNA expression of TNF-α and IL-6 **(B)** after TNF-α/IFN-γ challenge with or without EE that had been pre-incubated at 60, 80, or 100 °C for 30 min. **(C,D)** TEER **(C)** and relative mRNA expression of TNF-α and IL-6 **(D)** after TNF-α/IFN-γ challenge with or without EE that had been pre-digested with Proteinase K. **(E,F)** TEER **(E)** and relative mRNA expression of TNF-α and IL-6 **(F)** after TNF-α/IFN-γ challenge with or without EE that had been dialyzed using a 3.5 kDa MWCO membrane; the retentate (>3.5 kDa) and dialysate (<3.5 kDa) fractions were tested separately. The vehicle control, untreated EE, 100 °C heat-treated EE, Proteinase K-digested EE, and retentate and dialysate of EE were included as controls. Data are presented as mean ± SEM. *p < 0.05, **p < 0.01, ***p < 0.001, ****p < 0.0001; ns, not significant.

To investigate the nature of the bioactive component(s) in the earthworm extract (EE), we performed a series of characterization experiments. First, to exclude potential confounding effects from the extraction procedure itself, we generated a vehicle control sample produced under identical conditions but in the absence of earthworm tissue. As shown in Figure 10, this vehicle control exhibited no detectable protective activity in TNF-α/IFN-γ-challenged Caco-2 cells, confirming that the observed biological effects are specifically attributable to components from EE rather than to residual solvents, reagents, or non-specific matrix effects. Second, three independent approaches were employed to characterize the bioactive component(s): heat inactivation, protease sensitivity, and dialysis-based molecular weight fractionation. As shown in Figure 10, the protective activity of EE against TNF-*α*/IFN-γ-induced inflammation and intestinal epithelial barrier dysfunction in Caco-2 cells was completely abolished after heat treatment at 80 °C or 100 °C for 30 min (Figures 10A,B), as well as after Proteinase K digestion (Figures 10C,D). Dialysis fractionation using a 3.5 kDa MWCO membrane revealed that the protective activity was exclusively retained in the retentate (fraction containing molecules >3.5 kDa), while the dialysate (fraction containing molecules <3.5 kDa) exhibited no detectable activity (Figures 10E,F). Importantly, none of the above-treated EE samples (heat-inactivated, Proteinase K-digested, retentate, or dialysate) induced any adverse effects in Caco-2 cells when added alone (without TNF-α/IFN-γ stimulation), as evidenced by unaltered TEER values and baseline cytokine expression compared to control cells. Collectively, these results demonstrate that the bioactive component(s) in EE are proteinaceous, heat-labile, non-dialyzable macromolecules.

## 4 Discussion

Earthworms have been used since ancient times to clear heat, relieve spasms, dispel decay, and promote muscle growth. Studies have shown that EE can ameliorate autoimmune-related inflammatory diseases such as rheumatoid arthritis and asthma (25, 40). Given that UC shares similar autoimmune features, we hypothesize that EE has therapeutic effects on UC. Using a DSS-induced colitis model – which recapitulates key pathological features of human UC, including epithelial barrier disruption, immune dysregulation, and dysbiosis (41) – we demonstrated that EE treatment significantly ameliorated disease symptoms, including weight loss, diarrhea, hematochezia, mucosal damage, and inflammatory cell infiltration.

The UC patients and mice with colitis exhibit abnormal immune responses characterized by significant increases in proinflammatory factors such as TNF-α, IL-1β, and IL-6 (42, 43). TNF-α and IL-1β are endogenous mediators of the early process of inflammation; they increase the migration and phagocytosis of macrophages and neutrophils and promote the proliferation and differentiation of T cells, thus activating and amplifying inflammatory cascade signals, destroying intestinal barrier function and accelerating the development of intestinal inflammation (44–47). IL-6 regulate the proliferation and differentiation of T cells by activating signaling pathways such as the JAK/STAT3 and PI3K/AKT pathways, causing a sustained inflammatory response in the intestine (48, 49), which in turn promotes the development of tumors (50). Reports show that EE can exert anti-inflammatory effects in inflammatory diseases by regulating the TLR4/NF-κB pathway (25, 40). In this study, we found EE treatment significantly reduced the levels of TNF-*α*, IL-1β, IL-6, and p-p65 in UC mice, demonstrating its potent anti-inflammatory effect.

Impairment of the intestinal mucosal barrier is considered to be an important etiological factor in the development of UC (51). TJ proteins are important components of the mechanical barrier of the intestinal mucosa (9, 43). DSS is a polyanionic derivative of dextran that causes chemical damage to the intestinal epithelium due to the presence of sulfate groups, leading to a reduction in the expression of TJ proteins such as ZO-1 and Occludin, which can disrupt the epithelial barrier (41, 52). In this study, EE treatment significantly upregulated the expression of ZO-1 and Occludin in the colon tissues of mice and restores intestinal barrier integrity. LPS increases intestinal permeability by triggering an inflammatory cascade response to reduce the expression of TJ proteins, leading to intestinal barrier dysfunction (53, 54). To further investigate the direct protective effects of EE on the intestinal epithelial barrier, we employed an *in vitro* model of LPS-challenged Caco-2 cells. The results show that EE protects intestinal epithelial cells against LPS-induced barrier disruption via anti-inflammatory and junction-enhancing. Collectively, these findings indicate that EE restores intestinal barrier integrity by upregulating TJ protein expression.

Network pharmacology analysis predicted the PI3K/AKT pathway as a central target hub for EE, which was subsequently validated by both *in vivo* and in vitro experiments. EE significantly suppressed the activation of PI3K/AKT induced by DSS in mice and by LPS or TNF-α/IFN-*γ* in Caco-2 cells, without affecting basal pathway activity in healthy conditions, indicating a context-dependent therapeutic action. The essential role of PI3K/AKT inhibition was confirmed using the PI3K activator 740Y-P, which abolished EE-mediated protection against disease activity, histopathological damage, barrier dysfunction, and systemic inflammation. PI3K/AKT serves as a master regulator linking inflammation and barrier function: its activation promotes NF-κB nuclear translocation, leading to pro-inflammatory cytokine production, and simultaneously disrupts TJ protein assembly via phosphorylation of downstream effectors such as GSK-3β and AKT (55, 56). By inhibiting PI3K/AKT, EE concurrently suppresses NF-κB-mediated inflammation and enhances TJ-dependent barrier integrity, thereby addressing both pathological hallmarks of UC. In addition to PI3K/AKT, other pathways identified by KEGG enrichment, such as TNF and MAPK signaling, may also contribute to the therapeutic effects of EE, and their potential interplay with PI3K/AKT warrants further investigation.

In summary, EE demonstrates therapeutic potential against UC by targeting the PI3K/AKT signaling axis to suppress inflammation and restore intestinal barrier integrity. Nevertheless, several aspects warrant consideration for future studies. First, given the complexity of the crude extract, future research should focus on the isolation and identification of the active components responsible for the observed therapeutic effects. Second, all earthworms used in this work were of a single species (*Pheretima aspergillum*). Different *Pheretima* species may have compositional and bioactivity differences. Therefore, whether the protective effects of EE observed in this study are generalizable to other *Pheretima* species or earthworms from different geographic origins requires further investigation.

## 5 Conclusion

This study demonstrates that EE exerts significant protective effects against ulcerative colitis by targeting both inflammation and barrier dysfunction. Functionally, EE interrupts the pathological cycle of intestinal inflammation and barrier disruption by simultaneously suppressing NF-κB-driven cytokine release and upregulating key TJ proteins ZO-1 and Occludin. Mechanistically, we identified and validated the PI3K/AKT signaling pathway as a central therapeutic target of EE using an integrated network pharmacology and experimental approach. Notably, all efficacy was achieved without detectable short-term toxicity, underscoring EE’s favorable safety profile. Collectively, these findings provide a robust molecular foundation for advancing EE as a promising naturally agent for the management of inflammatory bowel disease.

## Data Availability

The original contributions presented in the study are included in the article/Supplementary material, further inquiries can be directed to the corresponding authors.

## Footnotes

1
https://tcmbank.cn

2
http://www.swissadme.ch

3
http://www.swisstargetprediction.ch

4
https://bioinformatics.psb.ugent.be

5
https://cn.string-db.org

6
https://www.genome.jp/kegg/

7
https://www.bioinformatics.com.cn
