# Supplementary material for: Earthworm extract ameliorates colitis via modulation of the PI3K/AKT pathway: attenuation of inflammation and restoration of intestinal barrier integrity
Source: Front Med (Lausanne). 2026 May 29;13:1787022. doi: 10.3389/fmed.2026.1787022 (PMC13259701; doi:10.3389/fmed.2026.1787022)
Supplement: Supplementary file 1 [file Supplementary_file_1.DOCX]

**Supplementary information**

**Supplementary Method**

**HPLC Analysis and Fingerprint Characterization of EE**

Chromatographic analysis was performed using reversed-phase high-performance liquid chromatography (RP-HPLC) on an Agilent 1260 Infinity II system equipped with a diode array detector. Separation was achieved on an Elite Hypersil ODS-2 column (4.6 × 250 mm i.d., 5 μm) maintained at 30°C. The mobile phase consisted of acetonitrile (A) and water (B) at a flow rate of 0.6 mL/min, with gradient elution as follows: 0–10 min, 1% A; 10–20 min, 1–3% A; 20–25 min, 3–8% A; 25–32 min, 8–20% A; 32–45 min, 20% A; 45–50 min, 20–50% A; 50–60 min, 50–100% A. The detection wavelength was 254 nm, and the injection volume was 10 μL. Reference standards for hypoxanthine, uridine, inosine, and guanosine (purity > 98%) were purchased from Shanghai Yuanye Bio-Technology Co., Ltd., and used for compound identification. For fingerprint analysis, raw chromatographic data from eight independently prepared batches of EE were imported into the Similarity Evaluation System for Chromatographic Fingerprint of Traditional Chinese Medicine (Version 2012, Chinese Pharmacopoeia Commission). The chromatogram of batch S1 was selected as the reference. Peak alignment was performed using the multipoint calibration method with a time window width of 0.1 min. Similarity coefficients between each batch and the reference fingerprint were calculated using the cosine method.

**Supplementary Figures**


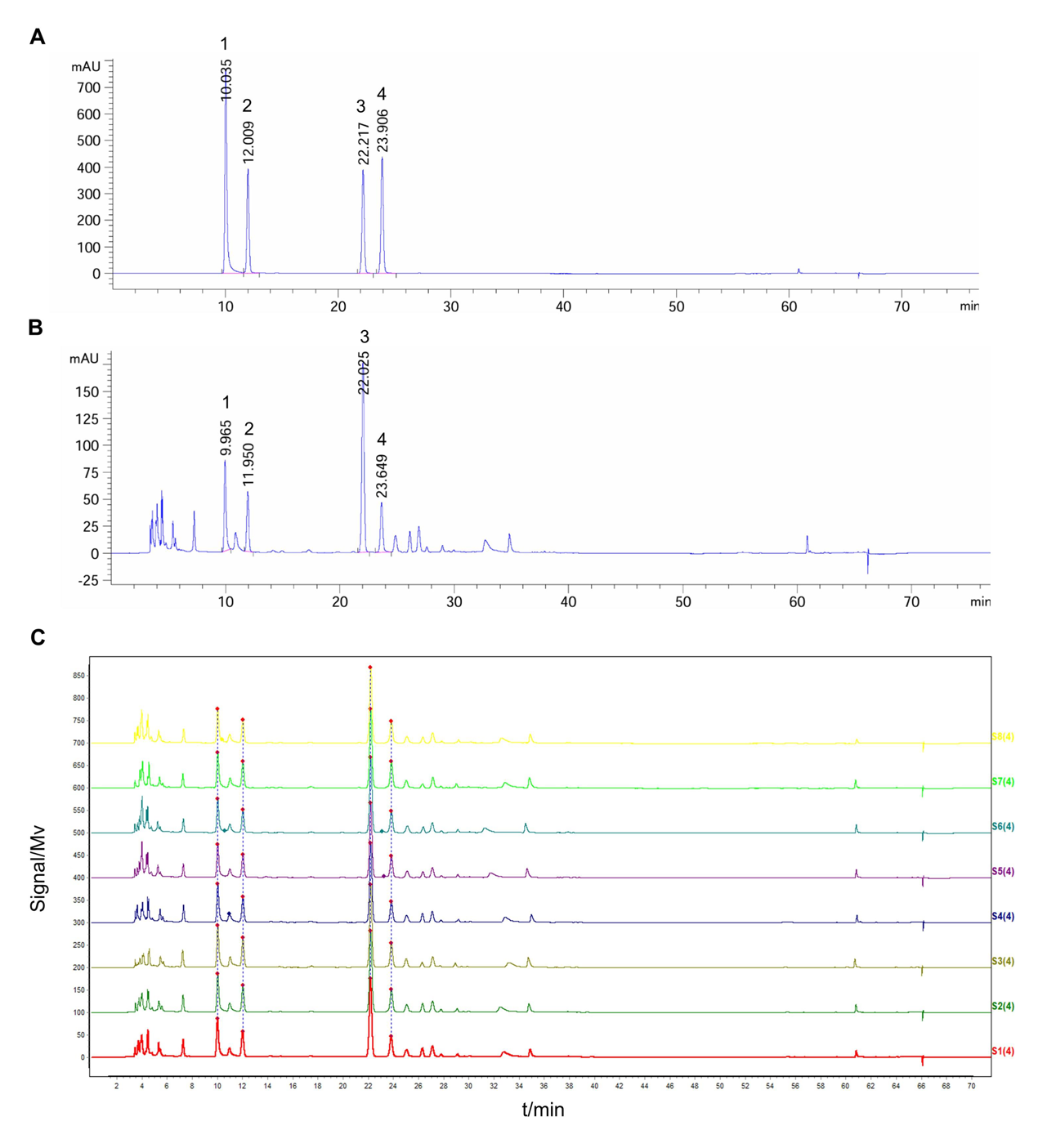
**Figure S1. HPLC analysis and fingerprint characterization of EE.** (A) Representative RP-HPLC chromatogram of mixed reference standards. Peaks: 1, hypoxanthine; 2, uridine; 3, inosine; 4, guanosine. (B) Representative RP-HPLC chromatogram of EE. The four characteristic peaks corresponding to the reference standards are labeled. (C) Fingerprint overlay of eight independently prepared batches of EE. The reference fingerprint was generated from batch S1, and all batches showed similarity coefficients greater than 0.99, indicating excellent batch-to-batch consistency.
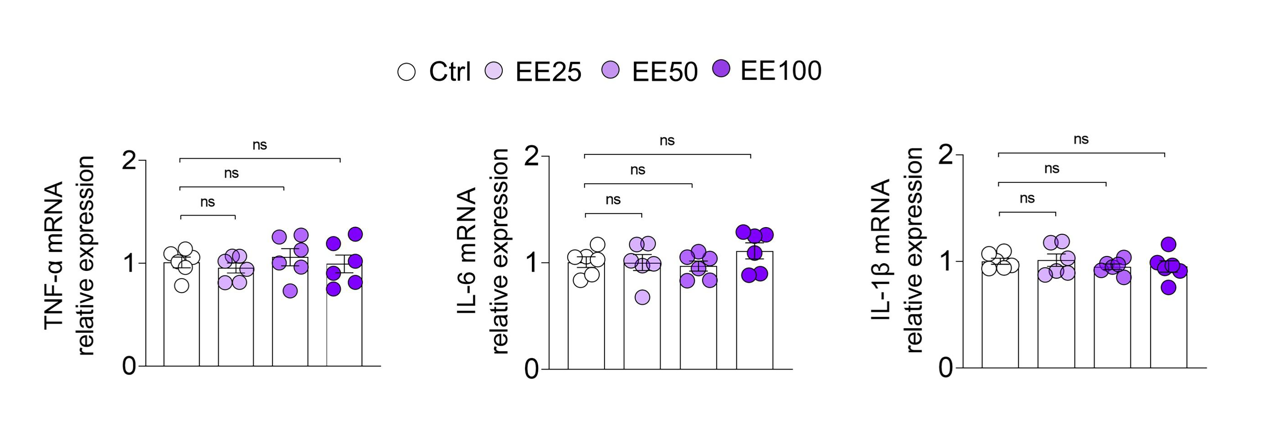
**Figure S2. EE alone does not induce pro-inflammatory cytokine expression in Caco-2 cells.** Caco-2 cells were treated with EE (25, 50, 100 μg/mL) for 24 h. The expression levels of TNF-α, IL-1β, and IL-6 were measured by qPCR. Data are presented as mean ± SEM. ns, not significant vs. Ctrl.

**Supplementary Table**

**Table S1.** **Endotoxin levels in eight independently prepared batches of EE.**

| Batch No. | Endotoxin Level (EU/mL) |
| --- | --- |
| 1 | 0.098 |
| 2 | 0.122 |
| 3 | 0.137 |
| 4 | 0.084 |
| 5 | 0.095 |
| 6 | 0.106 |
| 7 | 0.114 |
| 8 | 0.112 |
| Mean ± SD | 0.109 ± 0.017 |

Footnote: Endotoxin levels in eight independently prepared batches of EE were measured using the chromogenic LAL assay. Data represent the mean of duplicate measurements per batch. The mean ± SD of eight batches was 0.109 ± 0.017 EU/mL.
